# When Is “Pseudo–Ludwig’s Angina” Associated With Coagulopathy Also a “Pseudo” Hemorrhage?

**DOI:** 10.1177/2324709613492503

**Published:** 2013-06-10

**Authors:** Emily Lovallo, Sarah Patterson, Mitchel Erickson, Cynthia Chin, Paul Blanc, Timur S. Durrani

**Affiliations:** 1 Department of Emergency Medicine, Highland Hospital, Alameda County Medical Center, Oakland CA, USA; 2 University of California, San Francisco, CA, USA; 3 California Poison Control System, San Francisco Division, CA, USA

**Keywords:** Ludwig’s angina, hemorrhage, angioedema, brodifacoum, warfarin, sublingual hematoma

## Abstract

Sublingual hematoma secondary to short-acting anticoagulants such as warfarin has been labeled “pseudo–Ludwig’s angina” to distinguish it from the classic syndrome of localized infection and swelling involving the upper airway. Sublingual hematoma with airway compromise secondary to brodifacoum, a common long-acting anticoagulant rodenticide, has only been reported in the veterinary literature. We report a case of massive tongue swelling and impending airway compromise in the context of an intentional long-acting anticoagulant ingestion leading to coagulopathy. The swelling was initially presumed to be due either to infection or hemorrhage, but this was not supported by computed tomography scan imaging. Instead, the patient’s clinical course was consistent with corticosteroid-responsive angioedema, temporally associated with the ingested brodifacoum.

## Introduction

“Pseudo–Ludwig’s angina” is a descriptor that has been used for sublingual swelling due to noninfectious causes, as opposed to the true condition, Ludwig’s angina, which is a manifestation of regional suppuration in the neck. Multiple case reports have described sublingual hematoma secondary to hemorrhage in patients anticoagulated with warfarin.^1-9^ Sublingual hematoma with airway compromise secondary to brodifacoum, a common long-acting anticoagulant rodenticide, has only been reported in the veterinary literature.^10^ A large sublingual hematoma can mimic Ludwig’s angina; either etiology can compromise the airway. Initial treatment includes either reversal of the bleeding diathesis for the former or appropriate antibiotic coverage for the latter. Angioedema is yet another potential cause of upper airway obstruction due to swelling, but this etiology is not typically invoked as underlying “pseudo–Ludwig’s angina.” We report a case of massive tongue swelling and impending airway compromise in the context of an intentional ingestion of the long-acting anticoagulant brodifacoum. This presentation was initially suspected to be due to local hemorrhage, consistent with coagulopathy and the previously reported pattern of “pseudo-Ludwig’s angina” associated with warfarin misadventure. Ultimately, however, imaging excluded the presence of hematoma and the clinical course was more consistent with angioedema, temporally implicated in response to the brodifacoum ingestion.

## Case Report

A 32-year-old African American male-to-female transgender patient presented to the emergency department, complaining of inability to swallow and difficulty speaking for the previous 2 days. She denied odynophagia, fever, trauma, seizure, or significant respiratory symptoms. Past medical history included depression, chronic pain, and a remote suicide attempt. On physical exam, she was afebrile and had a normal respiratory rate and oxygenation by pulse oximetry. The oral examination was remarkable for a massively swollen tongue, elevation of the sublingual tissue, and fullness of the anterior neck. She could not vocalize. The remainder of the physical examination was unremarkable. The patient was given empiric intravenous clindamycin to cover for possible infection and dexamethasone to reduce swelling. Anesthesia and otolaryngology services were consulted for emergent airway management and the patient was taken directly to the operating room for fiber-optic nasal intubation. While in the operating room, prior to intubation, she communicated by writing that she had swallowed a rat poison 4 weeks prior to presentation in an intentional suicide attempt. Her initial international normalization rate (INR), which returned a short time later, was greater than 13.7.

During intubation, she was noted to have tongue, nasopharyngeal, oropharyngeal, left arytenoid, and epiglottic swelling. After intubation, she was admitted to the intensive care unit for management of her airway. To treat her coagulopathy, she was administered high-dose vitamin K and fresh frozen plasma. Dexamethasone and clindamycin were continued.

**Figure 1.**
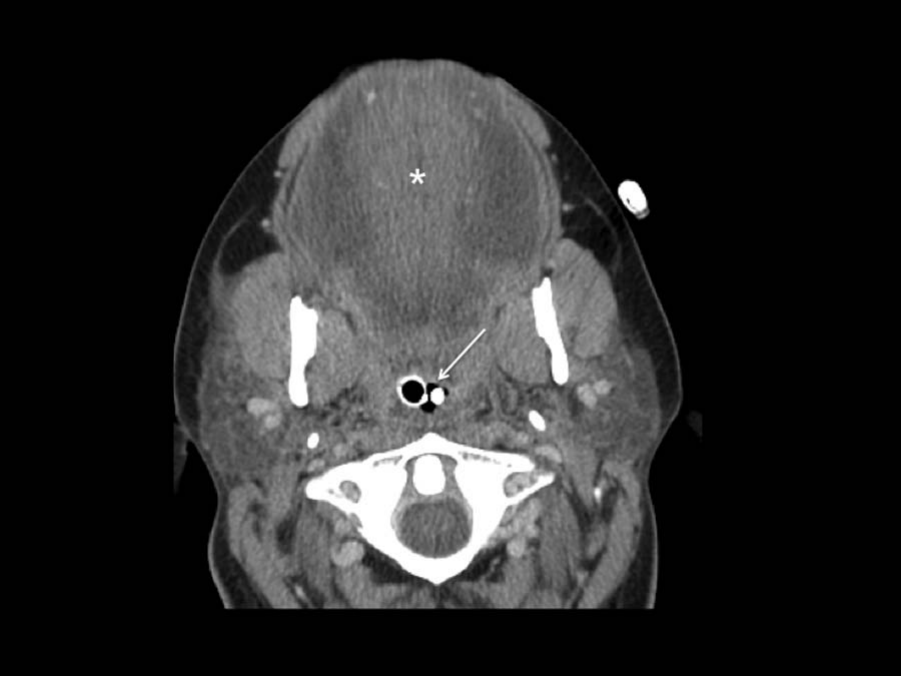
Axial contrast-enhanced computed tomography scan through the oropharynx demonstrates diffuse edema throughout the swollen tongue (*), which fills the entire oral cavity resulting in severe narrowing of the pharyngeal airway with patency maintained by endotracheal tube and nasogastric tube (arrow)

A computed tomography scan of the head was obtained on hospital day 2. This showed diffuse tongue, floor of mouth, submental, and submandibular space edema but no abnormal increased density that would be expected with a discrete hematoma or diffuse interstitial bleeding (Figure 1). Her airway swelling improved slowly and she was successfully extubated on hospital day seven. The patient was able to converse with minimal difficulty, being able to describe the brand packaging and price of the rat poison she ingested, which confirmed that it was a brodifacoum-containing product. She denied any co-ingestion or that she was taking any other daily medications at that time. The patient’s coagulopathy initially required vitamin K doses of 100 mg every 6 hours per feeding tube as well as intermittent fresh frozen plasma to maintain an INR less than 3. On hospital day 13, she was transferred out of the intensive care unit. Her corticosteroid therapy was tapered without recurrence of swelling. She was ultimately transitioned to vitamin K by mouth, which was safely reduced to a total daily dose of 25 mg daily. A psychiatric consultation determined that she was no longer a danger to herself and she was discharged on hospital day 17 with the plan to further taper her vitamin K by 2.5 mg every week and monitor weekly INR values. A brodifacoum level was not quantified.

## Discussion

This is the first case report of airway obstruction associated with over-anticoagulation yet without hematoma. Hematoma leading to such obstruction, referred to as “Pseudo–Ludwig’s angina,” is a well-established warfarin-associated phenomenon (including a case report from our own institution).^1-9^ It has only been documented previously in association with brodifacoum in the veterinary literature.^10^

The absence of hematoma, coupled with the clinical course, leads us to conclude that the patient’s presentation is best explained by angioedema and that this angioedema can be attributed to brodifacoum. Although angioedema is a well-recognized complication of many pharmaceuticals, it has neither been reported with anticoagulants nor is it listed in the package insert for Coumadin.^11^ Nonetheless, this potential complication of long-acting anticoagulants, even if unusual, is important because such ingestions are common. In 2010, there were 10 488 human exposures to similar long-acting anticoagulant rodenticides reported to US Poison Control centers, of which 2774 were treated in health care facilities.^12^

Similar to warfarin, brodifacoum inhibits the hepatic synthesis of vitamin K–dependent clotting factors. The addition of the brominated polycyclic hydrocarbon side chain to the basic warfarin structure has several properties that serve to promote anticoagulation: It allows brodifacoum to have a higher affinity for hepatic tissue, it interrupts the vitamin K(1)-epoxide cycle at more than one point, it has greater lipid solubility, and it manifests enterohepatic recirculation.^13^ Several of these attributes lead to a markedly longer half-life for brodifacoum as compared with warfarin.^14^ Management of hemorrhage secondary to overdose has been discussed in several studies.^15-21^

Bromine-containing drugs as a class have certain unusual features. Exposure to bromine and brominated products has been reported to cause a plaque forming dermatitis, termed “bromoderma.” The exact mechanism is unknown, but it is postulated to be either an inflammatory or delayed hypersensitivity reaction.^22,23^ This, however, is not a classic angioedema pattern. Nonetheless, it does underscore that bromine is associated with delayed responses and could signal a different risk profile than that of warfarin. Warfarin is not bromine containing and has been long and widely used without reported angioedema. Indeed, warfarin has been suggested as a potential treatment to prevent angioedema.^24-27^ Based on our review of the literature, we are unable to establish a clear mechanism of action for angioedema caused by brodifacoum. Angioedema is a well-recognized risk of angiotensin-converting enzyme (ACE) inhibitors.^28^ The chemical structure of brodifacoum, however, does not appear to be similar to that class of medications and there is no evidence to suggest that it functionally interacts with ACE receptors^29^ and it is not otherwise known to inhibit the metabolism of ACE.

We did not rechallenge the patient to brodifacoum or another bromine-containing substance to support a causal association, nor can we absolutely exclude that another unreported exposure may have been involved or perhaps interacted with the brodifacoum.

## Conclusion

Airway compromise secondary to over anticoagulation, although rare, previously has been well documented from local hematoma, rather than a result of the angioedema we report here. In addition to hemorrhage, airway angioedema should remain in the differential for the etiology of airway compromise, even when coagulopathy from an anticoagulant is present.
